# Supplementary material for: Why it is important to consider negative ties when studying polarized debates: A signed network analysis of a Dutch cultural controversy on Twitter
Source: PLoS One. 2021 Aug 31;16(8):e0256696. doi: 10.1371/journal.pone.0256696 (PMC8407581; doi:10.1371/journal.pone.0256696)
Supplement: S1 Appendix — (DOCX) [file pone.0256696.s001.docx]

Appendix for “Why it is important to consider negative ties when studying polarized debates: a signed network analysis of a Dutch cultural controversy on Twitter”

Section A: Issue Sentiment Classification

**Section A**: Issue Sentiment Classification

To analyze the debate on Black Pete, we want to know whether tweets in our dataset express a pro, anti, or neutral stance towards Black Pete. This *issue sentiment* is different from the general sentiment of the tweet, since a tweet with a negative tone of voice can be expressing a (positive) pro Black Pete statement (e.g. ‘Makes me so angry hea?!!!! Hands off of our tradition!!!! Let black pete be black!!!! Don’t make children cry!!!!! ’^[[1]](#footnote-1)^). Similarly, a tweet with positive tone of voice can be expressing a (negative) anti Black Pete sentiment (e.g. ‘Infinite respect for everyone that fought today for an inclusive sinterklaas celebration for everyone ❤️ #KickOutZwartePiet #kozp’^[[2]](#footnote-2)^. This implies that existing sentiment analysis algorithms cannot be used to identify the issue sentiment expressed in tweets. We therefore tailored a solution for the task at hand. This solution, a form of supervised machine learning, consists of two steps. First, we first manually classified the issue sentiment for roughly 5.300 unique tweets (2.7% of all unique tweet text) and manually identified the stance of the top prominent users. Second, we used these data as input for training a machine learning algorithm to classify the issue sentiment of the unlabeled tweets.

The issue sentiment of 5.300 unique tweets was labeled manually with the assistance of four fluent Dutch speakers. Each tweet was assigned one label: pro, anti, neutral or ambiguous. The codebook instructions were conservative: if the issue sentiment is not self-evident, the tweet was labeled as ambiguous. Table 1 below lists some examples of pro, anti, neutral, and ambiguous tweets in the data. The inter-coder agreement, measured by a Krippendorf Alpha of 0.72, was substantial. From the coding efforts we learned that it was often difficult to distinguish neutral from ambiguous tweets and we found few tweets (n=512) that were coded as expressing a neutral issue sentiment. Therefore, for further classification purposes, we merged the neutral and ambiguous tweets into one category. This manual classification identified 58% tweets with pro Black Pete sentiment, 13% tweets with anti-Black Pete sentiment and 30% tweets with neutral/ambiguous issue sentiment.

In addition to labeling this subset of tweets, we also manually labeled users’ issue stance for the users that belong to the 1% top-most frequently retweeted, mentioned and tweeting users by analyzing their tweets and their role in the media and the offline debate. Again, our codebook was conservative: if the stance of the user was not self-evident, the user was assigned an ambiguous stance towards Black Pete. Subsequently, the tweets of clear pro and anti-users (n=732) were classified as pro and anti respectively, increasing our classified tweet data to a total of 51.014 unique tweets.

Next, we applied a preprocessing pipeline to the labeled and unlabeled tweets to clean the data and prepare features for the machine learning. This pipeline consisted of: shortening urls to their main domain; transforming emoticons and emojis to strings; removing single characters, multiple spaces and linebreaks; substituting the @-sign followed by a username with ‘at_’ and the #-sign followed by a username with ‘hashtag_’; and substituting ‘...’ for ‘dotdotdot’ (since this has a particular meaning). We do not tokenize the text since this step is already integrated into the fastText algorithm.

To train the classifier, we split all labeled data into a training set (70%) and a test set (30%), ensuring that the test set contained no duplicates of the training set. Thereafter, we downsampled the training set to 3.000 pro, 3.000 anti, and 2.000 neutral tweets (the total of neutral tweets available) to ensure the classifier would not be biased towards a pro or anti sentiment.

Using the fastText algorithm and the labeled training data, a classifier was trained to classify the issue sentiment of tweets by maximizing the F1 score for all classes, thus attempting to predict all classes well, in both precision and recall. The fastText algorithm gives an indication of how certain the classification is (the softmax probability), valued between 0 and 1 for each prediction. We use this certainty indication to apply a simple rule: classify all tweets with lower certainty (<0.9) as neutral/ambiguous. This procedure reduces the recall for the pro- and anti-classes but also, more importantly, reduces the errors we care most about: classifying pro tweets as anti and classifying anti tweets as pro.

The classifier—after applying the certainty rule—categorizes the issue sentiments with sufficiently high accuracy; see Figure 1. There are only 28 cases in which an anti-tweet is misclassified as pro (0.012 times of all anti tweets and 0.16 times all pro tweet classifications) and 80 cases in which a pro tweet is misclassified as anti (0.007 times of all pro tweets and 0.13 times of all anti classifications). Classifying the full dataset, we find 15% anti tweets (n=65.314), 48% pro tweets (n=225.856) and 38% tweets with neutral/ambiguous issue sentiment (n=176.327).

Figure 1: The results of the classifier (parameter values: epoch=10, learning rate=0.7, n-grams=3) after applying the simple
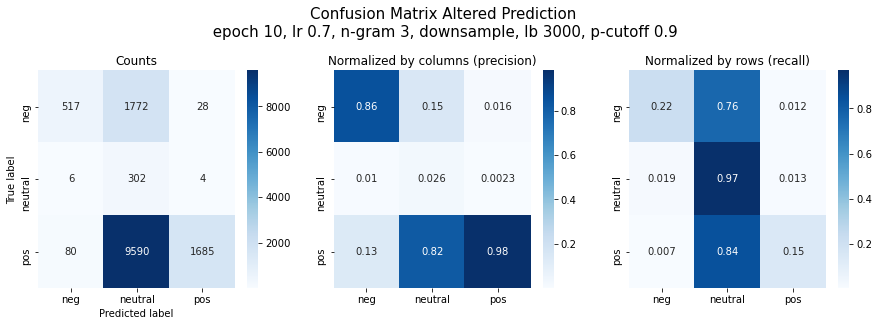
certainty rule (neutral if certainty < 0.9): confusion matrix with counts (left), normalized by the true labels (middle) and normalized by the predicted labels (right). The values in the diagonals of the middle matrix are the precision rates, and the values on the diagonals of the right matrix are the recall rates. Recall rates here are reduced due to the certainty rule, but the most important errors (classify positive if true value is negative and classify negative if true value is positive) are reduced.

| Tweet text Dutch | Tweet text English (author’s translation) | Labeled/ predicted |
| --- | --- | --- |
| Pro sentiment | | |
| Een grote meerderheid van Nederlanders kun je ook kwetsen @albertheijn Wij hebben ook gevoel! Stop met de afbraak van Nederlandse tradities zoals #zwartepiet en #Kerst. U verdient een #BoycotAH Twitteraars boos: geen ‘kerst’ maar ‘winter’ <https://t.co/zJ065VbUdR> via @telegraaf | You can hurt a large majority of the Dutch too @albertheijn We have feelings too! Stop with the destruction of Dutch traditions such as #blackpete and #Christmas. U deserve a #BoycotAH Twitterers angry: no ‘christmas’ but ‘winter’ <https://t.co/zJ065VbUdR> via @telegraaf | labeled |
| @LodewijkA Het enige doel van #KOZP is kinderen terroriseren. @LodewijkA heeft blijkbaar ook een hekel aan kleine kinderen. | @LodewijkA The only aim of #KOZP is to terrorize children. @LodewijkA apparently hates small children too. | predicted |
| Yak Laat zwarte piet gewoon zwarte piet blijven.!! Handen af van een kinderfeest.!! | Yikes Let black pete be black pete.!! Hands off of a childrens’ party.!! | predicted |
| @SylvanaSimons en die andere lamlul @TheRebelThePoet Hier een boodschap voor jullie #ZwartePiet https://t.co/e8n9f1PQXx | @SylvanaSimons and the other dump TheRebelThePoet Here a message for you #BlackPete https://t.co/e8n9f1PQXx | predicted |
| @NadiaBouras Huiliehuilie. Zwarte piet blijft toch!!! | @NadiaBouras Cry baby. Black pete stays anyway!!! | predicted |
| Anti sentiment | | |
| Zwarte Piet is racisme. | Black Pete is racism | predicted |
| Wat een raar frame? Anti-pietbetogers raakten in Rdam & Eindhoven niet ‘slaags’ met omstanders maar werden belaagd en bedreigd door honderden hooligans. In andere steden werden die opgepakt, niet de vreedzame activisten @NOS @Teletekst #falsebalance #kozp <https://t.co/vx9YPtoIVr> | What a weird frame? Anti-black pete supporters didn’t get into a fight in Rdam & Eindhoven with bystanders but were attacked and threatened by hundreds of hooligans. In other cities these were arrested, but not the peaceful activists @NOS @Teletekst #falsebalance #kozp <https://t.co/vx9YPtoIVr> | labeled |
| Wierd Duk fabriceert leugens over #KOZP in de krant, en een paar dagen later wordt #KOZP belaagd door hooligans en neonazi’s. Trots op jezelf, @wierdduk? <https://t.co/kvDhA7O8nv> | Wierd Duk creates lies about #KOZP in the paper, and a few days later #KOZP is attacked by hooligans and neonazi’s. Are you proud, @wierdduk? <https://t.co/kvDhA7O8nv> | labeled |
| De tirannie van de meerderheid wint in meer en meer steden. Gesteund door @Politie, @MinPres, politici en burgemeesters. Democratie? Vrijheid van meningsuiting? Gelijkheid in grondrechten? Waar? Nederland, je lelijkste gezicht is nu goed zichtbaar. #KOZP #ZwartePietIsRacisme https://t.co/ik7bFQYSnL | The tyranny of the majority wins in more and more cities. Supported by @Police, @Minpres, politicians and majors. Democracy? Freedom of speech? Equality in rights? Where? Netherlands, your most ugly face is now clearly visible. #KOZP #BlackPeteIsRacsim https://t.co/ik7bFQYSnL | labeled |
| #NeemAfstandVanWierdDuk #KOZP https://t.co/xG4Y4qGMMM | #DistanceYourselfFromWierdDuk #KOZP https://t.co/xG4Y4qGMMM | predicted |
| Hoi @jennydouwes @wierdduk @geertwilderspvv - Minder mensen kiezen voor traditionele Zwarte Piet \| NOS https://t.co/N5hbaVjTmw | Hoi @jennydouwes @wierdduk @geertwilderspvv – Less and less people are choosing for the traditional Black Pete \| NOS https://t.co/N5hbaVjTmw | predicted |
| Ambiguous or neutral sentiment | | |
| Beste pro- én anti zwarte piet demonstranten... HOU EENS OP MET DAT GEZEIK EN GA GEWOON EEN GEZELLIGE SINTERKLAASPERIODE MAKEN! | Dear pro- and anti black piet protesters... PLEASE STOP YOUR WHINING AND JUST GO HAVE A NICE SINTERKLAAS SEASON! | predicted |
| Sinterklaas is weer in het land. #weekend #Sinterklaas #sinterklaasintocht #Zwarte Piet https://t.co/vjyJ4oT88q https://t.co/wDnxyrJMoY | Sinterklaas is back in the country. #weekend #Sinterklaas #sinterklaasintoch #Black Pete https://t.co/vjyJ4oT88q https://t.co/wDnxyrJMoY | predicted |
| DENK wil Zwarte Piet de nek omdraaien https://t.co/qciqfh8nOw | DENK wants to kill Black Pete too https://t.co/qciqfh8nOw | predicted |
| @Boargemaster @DilanYesilgoz Onzin, die uitspraak kwam helemaal niet vanuit de KOZP hoek | @Boargemaster @DilanYesilgoz Nonsense, that statement didn’t come from the KOZP at all | predicted |
| in NL op straat tegen idioot hoge brandstofprijzen ??? nee man op straat voor of tegen zwarte piet.... massaal JA #prioriteiten lekker op n rijtje NL..... #koekoek | in NL on the streets against idiotic high fuel prices ???? No man in the street for or against black pete...massive YES #priorities straight in NL…. #cuckoo | predicted |
| De discussie over Zwarte Piet kabbelt voort, maar het draagvlak brokkelt langzaam af https://t.co/WS33B2aReJ via @volkskrant | The discussion about Black Pete ripples on, but support is slowly crumbling https://t.co/WS33B2aReJ via @volkskrant | labeled |

Table 1: Examples of tweets expressing a pro, anti or neutral/ambiguous sentiment on Black Pete. The column predicted/labeled indicates whether this tweet was classified by manual coding (labeled) in our dataset or predicted by the classifier (predicted).

1. This tweet was originally in Dutch and the link was here omitted for brevity: “https://t.co/vWND8ngJnT Word daar zo boos van he ?!!!!! Blijf van onze traditie!!!!! Laat zwarte piet zwart blijven!!!!! Laat kindjes alle sinds niet huilen!!!!!” [↑](#footnote-ref-1)
2. This tweet was originally in Dutch: “Oneindig respect voor iedereen die vandaag streed voor een inclusief sinterklaasfeest voor iedereen ❤️ #KickOutZwartePiet #kozp” [↑](#footnote-ref-2)
