# Supplementary material for: Why it is important to consider negative ties when studying polarized debates: A signed network analysis of a Dutch cultural controversy on Twitter
Source: PLoS One. 2021 Aug 31;16(8):e0256696. doi: 10.1371/journal.pone.0256696 (PMC8407581; doi:10.1371/journal.pone.0256696)
Supplement: Attachment — Submitted filename: Response to Reviewers.pdf [file pone.0256696.s003.pdf]

Dear Barbara Guidi,

Thank you for inviting us to resubmit the paper upon suitable minor revisions and giving us the opportunity to address the issues raised by the reviewers to our revised version. We wish to thank the reviewers for their close reading of the revised version of their constructive comments.

As specified in the decision letter, we have addressed the grammatical issues pointed out by reviewer 1, and this revised version of the article elaborates more details of the issue sentiment classification with the fastText algorithm, in accordance with the suggestions of reviewer 2. Additionally, we have uploaded an adjusted version of Figure 5, because we found a typo in this figure.

We hope you agree that our revision adequately addresses the concerns that the reviewers raised.

Kind regards,
the authors
