# Supplementary material for: Why it is important to consider negative ties when studying polarized debates: A signed network analysis of a Dutch cultural controversy on Twitter
Source: PLoS One. 2021 Aug 31;16(8):e0256696. doi: 10.1371/journal.pone.0256696 (PMC8407581; doi:10.1371/journal.pone.0256696)
Supplement: Attachment — Submitted filename: Response to Reviewers.docx [file pone.0256696.s002.docx]

Dear Barbara Guidi,

Thank you for considering our paper and for giving us the opportunity to address the issues raised by the reviewers and yourself.

You suggested we reconsider the title. We have now renamed the article to ‘Why it is important to consider negative ties when studying polarized debates: a signed network analysis of a Dutch cultural controversy on Twitter’ and hope that you agree this new title meets PLOS ONE’s title guidelines.

We wish to thank the reviewers for their close reading of the paper and their constructive suggestions. We have now revised the paper to address the reviewers’ concerns and we believe the structure and argument of the paper is much clearer. In this message, we explain how we addressed the major issues raised by the three reviewers, shown below on a point-by-point basis.

We have taken up the suggestion of all the reviewers to restructure the text and tighten the argument. We are grateful for this suggestion, because we believe that the article is now easier to read and brings out the main argument more clearly. As suggested by all reviewers, we have also had a professional proofreading service go through the article.

We hope you agree that our revisions adequately address the concerns that the reviewers raised.

Kind regards,

The authors

**Reviewer #1:** I thank the authors for this work that I am certain will be of interest to many given the subject matter of the debate and the authors' application of signed network analysis. For me the detail and nuance in the community definitions was the most appealing and informative part of this paper. I think there is some value in seeing the nature of participants and how they participate in this or other debates.

Dear Reviewer,

Thank you very much for your instructive feedback. We respond to all separate suggestions below.

I base my recommendation on potential to improve the presentation of content and organization, primarily in the introductory section of the paper. Following I provide some specific comments:

According to the PLOS publication guidelines, the Introduction is a required paper element and it precedes the “Materials and Methods” (Methods) section. To follow this guideline, I suggest the authors combine the current Introduction with the sections that follow and precede methods – perhaps by converting the headings, e.g., “Studying debates on Twitter” etc. to subheadings. I suggest the information about methods and results shown in the introduction as paragraphs two and three should be removed, so the introduction section is focused on providing the necessary background and review of literature to contextualize and rationalize the study.

Thank you very much for these suggestions. In accordance with them, we have restructured the introduction to include the literature review as a subsection. We have now collected all information on the case, data and methods into the ‘Materials and Methods’ section. This also allowed us to rewrite the text in such a way that the reader can smoothly transition from Introduction to Results, without necessarily reading the Materials and Methods section.

I also suggest the authors consider revising the phrase “The structure of our argument” to refer to this manuscript, i.e., “This paper is organized as follows” or “In the remainder of this paper, we begin by outlining…” I do not think “argument” is the best term for an empirical research report.

Thank you for pointing this out, we have changed the text accordingly.

Introduction – “Roughly one-third of social media users” – this is clearly stated in the cited report. I am not certain the other part of the sentence - “and social media has become one of the most important sources for learning about current political debates” - is supported in this report as broadly as the authors suggest. The cited report only speaks to social media users, so to me me this does not support “has become one of the most important sources” unless this is conditioned upon something like for instance, being a politically engaged social media used. I suggest the authors modify this statement as appropriate.

Thank you for this feedback. We have rewritten the text, please see page 2 and 3.

I have a similar response to the next sentence – I suggest the authors define, specify or offer a citation for “have become an important resource for social science research.” It is the assertion “important resource” that I question.

Thank you for this feedback. We have rewritten the text, please see page 2 and 3.

First paragraph in “Studying” section, second sentence: “These data have fueled” rather than “has”

Thank you for pointing this out. We have now changed all descriptions of prior research to past tense.

Second paragraph in “Studying” section: “Twitter, in particular, has been the preferred social media platform” – this assertion requires a citation. It is the word “preferred” that I think needs support.

Thank you for pointing this out. We have rewritten the text, please see page 2 and 3.

Same paragraph – it might be worthwhile to define “unsigned network analysis” if the intended audience is any social science researcher versus data scientists. Understanding this concept is important for readers to make sense of this work.

Thank you very much for this suggestion. We have taken your suggestion and defined unsigned networks in various places in the article: the abstract, introduction (second paragraph), and in the conclusion (second paragraph).

Top of third page describing prior research (Evolvi, Moernaut et al., Roy) – I believe prior research should be described using past, not present tense (Evolvi studied … and found…). This recommendation (use of past tense) also applies to other descriptions of prior research shown on this page.

Thank you for pointing this out. We have now changed all descriptions of prior research to past tense.

“Case” section: Santa Claus (no e) is the usual spelling for the character name, even though there is a film called "The Santa Clause."

Thank you for pointing this out.

“Case” section , paragraphs two and three: I recommend the authors provide citations to support the information about protests and activism. These might come from popular or news media or any other sources consulted by the authors to prepare this report

Thank you for this suggestion. We have now provided additional references to academic literature on the Black Pete debate. References to other sources, such as newspaper articles, can be found in these referenced works.

Methods section, paragraph four: Please describe to readers how the codebook used was designed and assessed.

Thank you very much for lifting this issue. Please see the text in the second paragraph of the ‘Issue and mention classification’ subsection, page 6. We have also added more details on the coding with example tweets in the appendix (‘Issue sentiment classification’).

Same section, paragraph five – please use past tense consistently in describing the research efforts (“counted,” “trained,” “provided,” “constructed”). This comment also applies to other sections of the methods that were written in present rather than past tense.

Thank you for pointing this out. We have now changed all descriptions of research efforts to past tense.

-------------------------------------------------------------------------------------------------------------

Reviewer #2: This is a review for “Why it is important to consider negative ties when studying polarized debates on Twitter”. The article used a dataset of the Twitter debate on Black Pete, covering the period from December 2017 to May 2019. In general, the manuscript would benefit from streamlining the overall structure and focusing the argument more tightly. Right now, the reader has to jump around to different concepts and methods, making the argument hard to follow. Focusing each paragraph on a clear topic sentence of sorts – “the main point” – and then unfolding any relevant citations and explanation in support would be useful, making sure that these citations are clearly relevant and tied to said topic sentence.

Dear Reviewer,

Thank you very much for your instructive feedback. We respond to all separate suggestions below each paragraph.

My main concerns are as follows:

1. Past tense should be paid attention in illustrating results.

Thank you for pointing this out. We have now changed all descriptions of previous research and illustrations to past tense.

2. The summarized results could be given in the abstract to attract readers’ interests and attention.

Thank you for pointing this out. We have rewritten the abstract to make the argument clearer, and we have summarized some specific results in the abstract to attract the readers’ interest and attention.

3. “Contention” as the keyword showed just once in this manuscript, which could be taken place by “trust building”.

Thank you for this suggestion. We have removed contention from the keywords since we only use its adjective form (contentious) throughout the article.

4. Definitions of polarized debates could be given out in second paragraph, since first paragraph was the background information. Given that this is the central construct under investigation in this study, the author should make sure that it is clearly and consistently defined and operationalized throughout the manuscript. Furthermore, the author should provide clear justification, via citations of previous research or otherwise, for why their definition is the most relevant and useful one to employ.

Thank you for this suggestion. We have included a definition of polarization in the second paragraph of the introduction and included references.

5. What is the drawbacks of considering only positive relationships. Giving the importance of introducing an approach for identifying positive and negative interactions in online debates. “However, while animosity…has considered only positive relationships” One sentence cannot explain the gap in the previous literature. The author need to present that the current research can fill the research gap of each previous literature.

Thank you for pointing this out. We have adjusted the text in the second paragraph of the introduction, page 2, to explain that we hypothesize that there are drawbacks in the current unsigned network analysis methodology. By comparing an unsigned with a signed analysis, we can pinpoint what those drawbacks are.

6. All the variables or instruments should be discussed in the introduction part. More importantly, review on variables of trust building items should be presented in the first part rather than in the discussion part.

All variables are now introduced in the introduction.

7. The introduction need more elaboration. I suggest rewriting these parts.

Thank you very much for pointing this out. In accordance with your suggestions, we have rewritten and restructured the introduction and the literature review subsection. We believe the argument is now much easier to follow for the reader.

8. Since the author mentioned social media, the effects of social bots could be considered. There are currently a handful of studies that analyze the effects of social bots. Most of these are within politically charged online conversations, including the United Kingdom’s Brexit referendum (Howard & Kollanyi, 2016), the ongoing Ukraine–Russia conflict (Hegelich & Janetzko, 2016), and the 2016 United States Presidential Election (Bessi & Ferrara, 2016).

Thank you for this suggestion. We agree that it would be very interesting to study the effect of bots in this dataset. However, this is beyond the scope of this particular research endeavor. We have looked into the most influential user accounts (being retweeted and retweeting/mentioning), and none of these are bots.

9. How to classify the issue sentiment of all tweets by the fastText algorithm?

Thank you for posing this question. We have now attached an appendix that explains in detail how the issue sentiment of tweets was categorized using manual labeling and the fastText algorithm.

10. Please list the serial number and name below the figures.

We understand this request and agree that it would make the review process more transparent. However, we cannot fulfil this request because we need to adhere to the guidelines of PLOS ONE for the figures , which indicates that the figures should be uploaded as separate files, without title, captions, or numbers.

<https://journals.plos.org/plosone/s/figures>

<https://journals.plos.org/plosone/s/submission-guidelines>

11. Potential multicollinearity problems should be tested with tolerance statistics.

Since we do not make claims on the impact of independent variables on dependent variables, we believe no problem of multicollinearity arises.

12. Some of the discussion of existing literature does not always seem relevant. This may be due to the argument getting muddled, or it may be that the authors can simply cut less relevant tangents.

Thank you for pointing this out. We have tightened the argument throughout the text, particularly in the introduction and literature section.

4. The discussion should be reorganized more logically. For example, the content of "Limitations and future research direction" could be added.

Thank you for pointing this out. We have added limitations and future research suggestions to the conclusion.

MINOR POINTS:

Some references list authors completely, others do not.

Thank you for pointing this out. We have made the reference list consistent.

The manuscript should be checked for APA style. Some grammatical errors making text hard to follow. Throughout the manuscript, there are a lot of language issues (e.g., article use of a and the, syntax). I would recommend having the paper proof read.

Thank you for pointing this out. We have sent the article to an editor.

-------------------------------------------------------------------------------------------------------------

Reviewer #3: Thanks authors for investigating such an important subject; I have the following comments:

Dear Reviewer,

Thank you very much for your instructive feedback. We respond to all separate suggestions below each paragraph.

There are some unnecessary details in the introduction such as an elaborate explanation about black Pete. I recommend summarizing the information about the debate and instead focusing on the method that is used to analyze such a debate and its comparison with previous methods.

Thank you for pointing this out. We have tightened the argument throughout the article and removed some of the details about the Black Pete tradition, see page 5. We have also restructured the ‘Materials and Methods’ section into subsections to allow reader-selected reading into specific parts of the case, data and methods deployed.

The Materials and method section is mixed with the Results, Discussion and Conclusions section. The materials and method section needs to be separated from other parts; only the Results, Discussion and Conclusions section can be combined.

Like the introduction, the method section has some unnecessary information and its combination with results and discussion makes it hard to follow the important points.

Thank you for pointing this out. We have rewritten the Materials and Method section to include solely information on the case, data, and methods. We have kept information on the case, data, and methods in the other sections to a minimum. We thank the reviewer for this suggestion, since we believe it is now much easier for the reader to smoothly transition from introduction to results and conclusion, without necessarily reading the methods section.

Figure 8 is not clear to read.

Thank you for pointing this out. We agree that the figure is very difficult to read. However, this is due to the markup of the PLOS ONe editorial process. Please click ‘click here to access/download;Figure:Fig8.tif’ on the top right of the page to download the original figure file that we have uploaded. This is of high quality and good to read.
